# Fiscal expenditure efficiency of China's coal to clean heating policy

**DOI:** 10.1016/j.heliyon.2024.e37621

**Published:** 2024-09-07

**Authors:** Fei Mo, Yaoyao Ren

**Affiliations:** a School of International Organizations, Beijing Foreign Studies University, 19 Xisanhuan North Road, Beijing, 100089, China; b Commodity Research Institute, China Risun Group Limited, Bldg. 1 Risun Plaza, 6 Sihezhuang Road, Beijing, 100070, China

**Keywords:** Coal to clean heating policy, Government subsidies, Data envelopment analysis, Slacks-based measure model with undesirable outputs, Five-dimension, 17-factor distribution model

## Abstract

Government subsidies are the backbone of the large-scale Coal to Clean Heating Policy launched in China in 2017. We analyze data from 27 pilot cities from 2017 to 2019. We use a slacks-based measure model with undesirable outputs to evaluate the optimal design of central and local government subsidies under the policy. While the overall efficiency is high, provincial capitals and cities with lower economic development levels show less efficiency. Our findings suggest that distributing central government subsidies based solely on administrative levels is not the best way to achieve perfect efficiency. Additionally, perfect efficiency at the city level may take years to achieve. A sweeping phase-out of government subsidies could lead to undesirable policy outcomes. Content analysis of normative documents from the six best-performing cities and six least efficient cities indicates that better-formulated legal and policy documents may contribute to higher efficiency. As lessons for designing and evaluating large-scale energy policies, we recommend considering 17 factors that could impact subsidy efficiency. We also suggest implementing a progressive phase-out schedule as a measure of policy efficiency and identifying factors that could ensure the long-term success of nationally implemented government subsidies.

## 1 Introduction

As the largest consumer of coal globally [1], China has faced severe air quality deterioration and increased mortality rates due to coal-fired winter heating systems in northern China [2]. To address these problems, China introduced the Coal to Clean Heating Policy (CCHP) as part of its 2013 Air Pollution Prevention and Control Action Plan [3]. The policy was launched in 2017 through several central administrative documents [[4], [5], [6], [7]]. It aimed to replace winter coal consumption in northern China with clean energy sources (see Table A2 in Appendices for clean heating technology paths). The policy set ambitious targets: increasing the clean heating rate in northern China to 50 % by 2019 and 70 % by 2021, and achieving 90 % clean energy heating in the “2 + 26” key cities by 2019 and 100 % by 2021. The “2 + 26” cities include Beijing and Tianjin, two municipalities directly under the central government, and 26 cities in Hebei, Shanxi, Shandong, and Henan Provinces. These provinces are along the air pollution transmission pathway in the Beijing-Tianjin-Hebei Region.

Since its implementation, CCHP has significantly improved air quality [8,9], primarily due to government subsidies that incentivize the transition to clean energy [9,10]. Efficient allocation of these subsidies is crucial to ensuring the policy's effectiveness and affordability for residents, especially those with limited income. By the end of 2021, over 7 million households had switched to clean heating, achieving a clean heating rate of 73.6 % [[11], [12], [13], [14]]. Despite requests from four central government ministries for a mid-term performance evaluation of CCHP in the “2 + 26” key cities (Target Cities) [15], the city-level evaluation results have not been made public. Only a few Target Cities officially reported their progress, such as Jiaozuo and Xinxiang in Henan Province [16,17]. The lack of specific data on the clean heating rate in the Target Cities reveals a gap in evaluating these cities' performance.

This study addresses this gap by analyzing the efficiency^2^ of government subsidies in the Target Cities from 2017 to 2019 using the slacks-based measure model with undesirable outputs. Beijing, not included as a pilot city until 2021, is excluded from this analysis (see 3.2.1 for more information on data). Government subsidies, both central and local, are crucial for the success of CCHP. According to the original design [5], central government subsidies were distributed to pilot cities based on administrative levels. Municipalities directly under the central government would receive 1 billion Chinese Yuan (CNY) or approximately 137.6 million United States Dollars (USD) per year. Provincial capital cities would receive 700 million CNY (96.3 million USD) per year. Prefecture-level cities would receive 500 million CNY (68.8 million USD) per year. The central funding for each pilot city continues for three years. In 2021, the central government extended funding for air pollution prevention and control, including central government subsidies for CCHP, until 2025 [18]. Furthermore, the allocation of central government subsidies for CCHP changed from a prescribed standard to a competitive evaluation process without specific criteria [18]. Local government subsidies varied across regions, typically phasing out over several years, as seen in Hebei Province's three-year phase-out schedule with a year-to-year reduction rate of 50 % [9]. Positive environmental changes from CCHP have been documented in several studies [19,20]. But the efficiency of the current subsidy design in achieving positive outcomes remains an open question. This study evaluates whether the current subsidy design efficiently achieves CCHP's goals.

Our contributions to the literature are threefold. First, we provide a comprehensive analysis of the efficiency of government subsidies in the Target Cities, an area that has been largely overlooked in previous studies. Second, our study introduces a holistic subsidy distribution model that considers socio-economic conditions and proposes a more progressive phase-out schedule. This model addresses the identified gaps in the existing subsidy allocation process and provides a framework for optimizing resource allocation. Finally, we present policy recommendations based on our findings, highlighting the uneven efficiency profiles of government subsidies. Our recommendations aim to enhance the effectiveness of future subsidy allocations, ensuring alignment with both environmental goals and fiscal sustainability.

The insights from this study can inform evidence-based policies and government strategies to combat air pollution and promote a more sustainable and cleaner energy future for China. Additionally, our methodology and findings can guide the design and evaluation of government subsidies in large-scale energy policies globally, contributing to the broader discourse on sustainable energy transitions.

The remainder of this paper is organized as follows: Section 2 reviews relevant literature; Section 3 describes the data and methodology; Section 4 presents the results; Section 5 discusses the findings; and Section 6 concludes with lessons learned.

## 2 Literature review

In terms of the effect of energy policies on society, studies conducted in various countries have shown that large-scale energy policies play an important role in environmental governance and energy conservation. For example, Clancy et al. [21] reported that implementing a coal ban in Dublin, Ireland reduced respiratory diseases and cardiovascular deaths, significantly decreasing pollutant emissions. Similarly, Budya et al. [22] found that the conversion from kerosene to liquid petroleum gas in Indonesia succeeded after removing kerosene subsidies. Zhang et al. [23] demonstrated that China's policy approach to renewable energies has fostered the development of the renewable energy industry and related sectors with long-term strategic significance. Sadiq [24] conducted a cost-benefit analysis of the solar water heater system as a substitute for electric power and natural gas for water heating in the residential sector of Pakistan.

Regarding CCHP specifically, existing literature has primarily focused on the policy's effects and public satisfaction. In evaluating the policy's effects, researchers have used various methods, including econometric models, cost-benefit analysis, and life cycle assessment, to assess the policy's impact on environmental, economic, and health domains. Liu et al. [25] used a first-difference model based on a panel dataset of 16 districts in Beijing to show significant improvements in air quality due to the coal-to-gas policy. Using life cycle assessment and life cycle cost methods, Li et al. [26] analyzed the environmental impact and economic costs of household energy usage for the entire year under five scenarios before and after the coal-to-gas conversion. Studies have also examined public satisfaction with the policy as both heat users and beneficiaries. Hao et al. [27] conducted a survey to assess the satisfaction of residents in Hengshui (one of the 10.13039/100004791Target Cities) with CCHP and concluded that optimization of the subsidy scheme was needed due to the high demand for funds. Xu and Ge [28] used a resident satisfaction model (RSM) and survey data to evaluate the effectiveness and sustainability of the coal-to-gas policy in northern China. They found that overall satisfaction among residents was moderate. Chen and Kim [29] argued that public acceptance should be considered when providing economic support for technological transitions, particularly in policies involving long-term social obligations, such as fair cost-sharing and transparent decision-making. A few studies have briefly mentioned the need for optimal government subsidies to achieve CCHP's goals. Chen et al. [30] found that high subsidies exacerbate the government's fiscal deficit while undermining the sustainability of clean heating. Feng et al. [31] suggested that the uneven distribution of subsidies across different city governments increased the heating burden on low-income households and led to heating poverty in less developed regions. Xie et al. [32] found that replacing coal with electricity and gas increased energy poverty in multiple dimensions, while replacing it with clean coal decreased energy poverty. Meng et al. [8] reported a significant decrease in premature deaths attributable to residential emissions, indicating the policy's effectiveness but also identifying issues of inequality and inequity in the distribution of benefits and costs. But none have conducted a comprehensive evaluation of the efficiency of the subsidies at the city level. Table 1, Panel A, provides a summary of the studies on CCHP.

**Table 1:** Literature review.

**Panel A.** Previous assessment of CCHP.		
Author	Method	Summary
Liu et al. [25] Chen and Kim [29] Feng et al. [31]	DID and first-difference model; Energy, economic, and social analysis; Cost-benefit analysis	Test of policy effectiveness
Li et al. [26]	Life cycle assessment and life cycle cost analysis	Benefit analysis from capital investment, operation to termination
Hao et al. [27]	Questionnaire and research	Public satisfaction with policy
Xu and Ge [28]	RSM	Effectiveness and sustainability
Chen et al. [30]	Real options approach	Economic and environmental benefits of CCHP
Meng et al. [8]	Simulation	Environmental and health benefits of CCHP

**Panel B.** Research on the efficiency of funds use.		
Author	Method	Research questions
Wang [33]	DEA (VRS)	Governmental research funding efficiency of universities
Coupet [34]	DEA (Two-stage DEA)	Government funding and efficiency in nonprofit colleges
Sun et al. [35]	DEA (VRS)	Local per capita finance in China
Wen et al. [36]	DEA (VRS)	Government finance under the policy of supporting agriculture
Li [37]	DEA (VRS)	Investment in environmental governance
Sun and Galagedera [38]	DEA (VRS)	Efficiency of superannuation funds
Marschall and Flessa [39]	DEA (Two-stage DEA)	Efficiency of health care facilities
Du et al. [40]	DEA (CRS)	Allocation of fixed cost and resources
Dębkowska et al. [41]	DEA (CRS)	Efficiency of utilization of public funds for climate neutrality
Gao et al. [42]	DEA (SBM-undesirable)	Tax policy and total factor carbon emission efficiency
Lu et al. [43]	DEA (SBM-undesirable)	Urban green innovation efficiency in China
Wang et al. [44]	DEA (SBM-undesirable)	Ecological welfare performance and green economic efficiency

Our study makes two notable contributions to the existing literature. First, we address a research gap by conducting a comprehensive analysis of the efficiency of government subsidies in the Target Cities, an area that has been largely overlooked in previous studies. Second, our research provides valuable insights for policymakers and planners concerning CCHP and other large-scale energy policies. We recommend developing a holistic subsidy distribution model for central government subsidies. We also suggest implementing a more progressive phase-out schedule to enhance the affordability and sustainability of clean heating interventions. Additionally, we advise adopting a cohesive approach to shaping local normative and policy documents that align with the central policy design. These suggestions aim to facilitate smoother and more effective policy implementation, ultimately leading to improved outcomes in the transition toward cleaner and more sustainable heating systems.

Data envelopment analysis (DEA) models have been widely used to evaluate fiscal expenditure efficiency, the main focus of this study. Wang [33] used the DEA method to evaluate the performance of financial funds allocated by the Chinese government to universities, developing a performance-based approach for a central planner to allocate research funding to different universities to stimulate better research output. Similarly, Coupet [34] employed a two-stage DEA model to measure the efficiency of private nonprofit teaching-oriented colleges and examined the impact of federal and state funding on organizational efficiency. Sun et al. [35] used the DEA-Tobit model to calculate the fiscal expenditure efficiency of 30 provincial and local governments in China in 2013. They took the per capita fiscal expenditure as the input variable and the government performance under the Five Major Development Concepts as the output variable and investigated the influencing factors. Wen et al. [36] applied the DEA model to evaluate the efficiency of fiscal support policies for agriculture in 30 provinces and cities in China to promote urban-rural economic integration from 1997 to 2015. Li [37] applied the DEA model to study the investment efficiency of environmental pollution control in China from 2000 to 2012. In view of the management of superannuation funds, Sun and Galagedera [38] introduced disbursement utilization and risk utilization under the DEA methodological framework to compare the performance of disbursements and risk management in overall management performance. Marschall and Flessa [39] applied the DEA method to evaluate the efficiency of healthcare facilities in Africa and proposed solutions to address serious shortages of personnel, medical supplies, and financial funds. Du et al. [40] developed a DEA-based iterative approach to allocate fixed costs and resources. Dębkowska et al. [41] proposed an approach based on the Data DEA method and soft modeling to assess the efficiency of public fund utilization for climate neutrality. Some studies used the slacks-based measure (SBM) model with undesirable outputs, the method applied in this study. For example, Gao et al. [42] used the value added tax reform in China as an exogenous shock and the SBM model with undesirable outputs to measure the total factor carbon emission efficiency of 282 cities in China. Lu et al. [43] established a framework using the model for assessing urban green innovation efficiency. Wang et al. [44] employed the model to evaluate the ecological welfare performance and green economic efficiency of 11 cities in China. Table 1, Panel B, summarizes the studies using DEA models to measure fund use efficiency.

## 3 Materials and methods

### 3.1 Methods

DEA is a widely-applied benchmarking method that can measure the efficiency of a decision-making unit (DMU) in a relative sense by comparing its performance against other DMUs [45]. The model can mitigate the subjectivity of decision-makers to a certain extent and is capable of dealing with DMUs with multiple inputs and multiple outputs. The model is now widely used in performance and efficiency evaluations in many fields [46].

As a non-parametric evaluation method, DEA has evolved into more than 140 models. In this article, we use the SBM model with undesirable outputs to solve the research problem. We note that there are different DEA models for undesirable outputs, such as the hyperbolic model (HYP model) [47], directional distance function model (DDF model) [48], undesirable output as input model (UINP model) [49], Seiford and Zhu model (SZ model) [50] and SBM model [51]. The advantages and disadvantages of the aforementioned models have been extensively investigated [52]. The SZ model relies on a specific translation vector defined before computation, and its accuracy could be compromised if the translation vector is altered. Moreover, no universally accepted procedure exists for selecting the translation vector for the SZ model. The UINP model treats undesirable outputs as inputs and has been criticized for its lack of conceptual soundness. Finally, the DDF and HYP models could be competitive for large samples. However, our study involves a relatively small sample size of 27 Target Cities. Therefore, the SBM model with undesirable outputs is preferred as a reliable alternative. The SBM model has demonstrated excellent performance for desirable outputs [53]. It is a non-oriented model that allows inefficiency to be measured from both input and output perspectives. It includes a slack improvement component in addition to the equal-proportional improvement component. Consequently, the SBM model overcomes the limitation of the radial model, which does not incorporate a slack variable in its measure of inefficiency. Based on previous studies, we have chosen the SBM model with undesirable outputs for our analysis.

#### 3.1.1 SBM model

The traditional DEA model is a radial model that measures the efficiency level by proportionally changing a DMU's inputs and outputs. But this may not be sufficient for inefficient DMUs, which include not only equal proportion improvement but also the improvement of the slacks. Slacks refer to the difference between actual and the ideal values for units on the efficiency frontier. Therefore, Tone [51,54] proposed the SBM model. In the SBM model, ρ represents the efficiency of the evaluated DMU. When ρ equals 1, the DMU is efficient; otherwise, it is inefficient. Let $X=\left(x_{1},x_{2},\ldots ,x_{n}\right)\in R^{m\times n}$ denote the matrix of all inputs, where $x_{i}$ is a column vector of *m* elements. Let $Y=\left(y_{1},y_{2},\ldots ,y_{n}\right)\in R^{q\times n}$ denote the matrix of all outputs, where $y_{i}$ is a column vector of q elements.

The calculation formula for the SBM model is as follows:

$$\rho =\min\;\frac{1-\frac{1}{m}\sum_{i=1}^{m}s_{i}^{-}/x_{io}}{1+\frac{1}{q}\sum_{r=1}^{q}s_{r}^{+}/y_{ro}}$$

(1) $$s.t.\;X\lambda +s^{-}=x_{o}$$

$$Y\lambda -s^{+}=y_{o}$$

$$\lambda \geq 0，s^{+}\geq 0，s^{-}\geq 0$$

The SBM model in (1) is a fractional programming problem because slack variables appear in both the denominator and numerator. The term $1/m\times \sum_{i=1}^{m}s_{i}^{-}/x_{io}$ represents the average relative gap between DMU o and the best-practice frontier on all inputs. Similarly, the term $1/q\times \sum_{r=1}^{q}s_{r}^{+}/y_{ro}$ represents the relative gap between o’s outputs and the frontier. A DMU is efficient when all slacks equal zero.

#### 3.1.2 SBM-undesirable model

In real-world production or project operations, final output does not always follow the logic of “the more, the better.” Undesirable outputs, such as air pollutants and wastewater, may arise. Administrators aim to minimize those undesirable outputs while maximizing desirable ones. Traditional DEA models are inadequate when undesirable outputs are present because they aim at increasing outputs. The SBM model can be adapted to account for undesirable outputs, making it suitable for environmental or energy studies. For example, one study evaluated the green innovation performance of Chinese pollution-intensive industry from 2014 to 2018 from two dimensions of transformation efficiency (static) and productivity (dynamic) using the SBM model with undesirable outputs and the Malmquist–Luenberger productivity index [55]. Another study used the SBM model with undesirable outputs to explore China's provincial energy efficiencies and meta-technology ratios of eight major economic regions from 2000 to 2014 [56]. The results show that there is a serious regional imbalance in China's energy efficiency. Another study used the imprecise DEA model and undesirable outputs to develop the WPF-SBM model [57]. They then applied the proposed model to a supplier selection problem. A numerical example demonstrates the application of the proposed model.

We assume there are $q_{1}$ desirable outputs and $q_{2}$ undesirable outputs. We separate the output matrix into the desirable matrix $Y^{g}=\left(y_{1}^{g},y_{2}^{g},\ldots ,y_{n}^{g}\right)\in R^{q_{1}\times n}$ and the undesirable matrix $Y^{b}=\left(y_{1}^{b},y_{2}^{b},\ldots ,y_{n}^{b}\right)\in R^{q_{2}\times n}$. Similarly, $s^{g}$ is the vector of $q_{1}$ slacks for desirable outputs, and $s^{b}$ is the vector of $q_{2}$ slacks for undesirable outputs. The SBM model with undesirable outputs is formulated as follows:

$$\rho =\min\;\frac{1-\frac{1}{m}\sum_{i=1}^{m}s_{i}^{-}/x_{io}}{1+\frac{1}{q_{1}+q_{2}}\left(\sum_{r=1}^{q_{1}}s_{r}^{g}/y_{ro}^{g}+\sum_{r=1}^{q_{2}}s_{r}^{b}/y_{ro}^{b}\right)}$$

(2) $$s.t.\;X\lambda +s^{-}=x_{o}$$

$$Y^{g}\lambda -s^{g}=y_{o}^{g}$$

$${\;Y}^{b}\lambda +s^{b}=y_{o}^{b}$$

$${\;s}^{-}\geq 0,s^{g}\geq 0,s^{b}\geq 0,\lambda \geq 0$$

The only difference between the SBM model (1) and (2) is that the undesirable slack $s^{b}$ has a positive sign “+” in front of it in model (2). However, model (2) is still a fractional model. To convert it into a linear model, the slack variables need to be moved out of the denominator. Using the Charnes-Cooper transformation, model (2) can be transformed into and solved as a linear program, as shown in model (3).

$$\rho =\min\;t-\frac{1}{m}\sum_{i=1}^{m}\frac{S_{i}^{-}}{x_{io}}$$

(3) $$s.t.\;t+\frac{1}{q_{1}+q_{2}}\left(\sum_{r=1}^{q_{1}}S_{r}^{g}/y_{ro}^{g}+\sum_{r=1}^{q_{2}}S_{r}^{b}/y_{ro}^{b}\right)=1$$

$$X\Lambda +S^{-}={tx}_{o}$$

$$Y^{g}\Lambda -S^{g}={ty}_{o}^{g}$$

$$Y^{b}\Lambda +S^{b}={ty}_{o}^{b}$$

$$\Lambda \geq 0,s^{-}\geq 0，s^{g}\geq 0，s^{b}\geq 0$$

Where $\Lambda =t\lambda ,S^{-}={ts}^{-},S^{g}={ts}^{g}\;and\;S^{b}={ts}^{b}$.

### 3.2 Materials

#### 3.2.1 Data

We collected financial information from various governmental departments at the central and local levels. The sources include the local bureaus of finance and ecology and environment of the Target Cities, the Ministry of Housing and Urban-Rural Development, and the National Energy Administration. The annual financial accounts of each Target City provided data on local financial expenditures for clean heating. Due to inconsistencies in statistical methods, we could not obtain complete information on the specific fiscal expenditures for CCHP in all Target Cities. As CCHP is a significant measure under the air pollution control policy [5,7,58,59], we selected the line item “air pollution” in the annual financial accounts. We used the ratio of air pollution expenditure to the local population, namely, per capita air pollution expenditure, as proxy for local fiscal expenditure on CCHP. We adopted the per capita expenditure to accurately represent the intensity of local financial subsidies, following previous research [60,61]. Additionally, we obtained the amounts of central financial subsidy for clean heating from the annual financial accounts of the Target Cities. We also gathered information on economic development and population from socio-economic development announcements, government work reports of each Target City, among others.

According to official information, Beijing was not included in the pilot cities receiving central government funds until the fourth batch released in 2021 (for a full list of the pilot cities, see Table A1 of the Appendices). Therefore, the data for Beijing from 2017 to 2019 may not reflect the efficiency of government subsidies under CCHP. As a result, Beijing, although a Target City, was excluded from this study.

The impact of implementing the clean heating policy focuses mainly on reducing air pollutants. To measure this, we utilized the daily average concentrations of PM2.5, SO_2_ and NOx for each Target City obtained from the China Air Quality Online Monitoring and Analysis platform, a public interest software platform [62].

#### 3.2.2 Variables

We employed two input variables. The first, “Local financial subsidy for air pollution per capita,” refers to the ratio of the amount of “air pollution” in the annual final accounts of the local bureaus of ecology and environment to the city population. We chose the “air pollution” line item to signify the level of local investment in financial funds for CCHP. While CCHP was not specifically listed in the financial accounts, it represents most of the investment in air pollution control. The second input variable, “Central financial subsidy for clean heating,” refers to the financial subsidy issued by the central government to a city.

We utilized four output variables. The first, “New area using centralized heating,” refers to the year-to-year increase in the area where heating is provided through centralized heating sources. The other three output variables, “PM2.5,” “SO_2_,” and “NOx,” represent the emissions of particulate matter less than 2.5 μm, sulfur dioxide, and nitrogen oxides, respectively. Table 2 provides an overview of all variables.

**Table 2:** Summary of variables.

Inputs-Outputs		Variable	Symbol	Meaning or Measurement	Unit
Inputs	Local subsidy	Local financial subsidy for air pollution per capita	$x_{1}$	Ratio of the amount of “air pollution” in annual final accounts of local bureaus of ecology and environment to population of the city	CNY
	Central subsidy	Central financial subsidy for clean heating	$x_{2}$	Financial subsidy issued by central government to a city	10,000 CNY
Outputs	Desirable outputs	New area using centralized heating	$y^{g}$	Year-to-year increase in the area where heating is provided through centralized heating sources	10,000 m^2^
	Undesirable outputs	PM2.5	$y_{1}^{b}$	Emission of particulate matter less than 2.5 μm in diameter	ug/m^3^
		SO_2_	$y_{2}^{b}$	Amount of sulfur dioxide emitted	ug/m^3^
		NOx	$y_{3}^{b}$	Amount of nitrogen oxides emitted	ug/m^3^

We selected these variables for several reasons. In implementing CCHP, both the central and local governments provide financial subsidies to the Target Cities. These financial funds are used by the 10.13039/100004791Target Cities to carry out clean heating projects, including technical pathways such as “coal to gas,” “coal to electricity,” and the energy-saving transformation of existing buildings. The expected results include increased areas utilizing centralized heating in the Target Cities and reduced undesirable environmental consequences. The two input variables, “Local financial subsidy for air pollution per capita” and “Central financial subsidy for clean heating,” represent financial subsidies from local and central governments, respectively. Among the four output variables, “New area using centralized heating” reflects the benefits of CCHP, while “PM2.5,” “SO_2_,” and “NOx” reflect the reduction in negative environmental consequences resulting from the policy. This study aims to explore the efficiency of fund utilization under CCHP, covering both central and local government funds. Furthermore, considering that the primary objectives of CCHP involve air pollution control and renewable energy development, measuring air pollutant concentrations is crucial to reflect the policy's impact.

We also conducted a correlation analysis of the chosen variables (Table 3). The results demonstrate correlations between the input variables $x_{1}$ (Local financial subsidy for air pollution per capita) and $x_{2}$ (Central financial subsidy for clean heating) and the output variables, namely, $y^{g}$ (New area using centralized heating), $y_{1}^{b}$ (PM2.5), $y_{2}^{b}$ (SO_2_), and $y_{3}^{b}$ (NOx). Specifically, $y^{g}$ exhibits a positive correlation with $x_{1}$ and $x_{2}$, while $y_{1}^{b}$, $y_{2}^{b}$ and $y_{3}^{b}$ display a negative correlation with $x_{1}$ and $x_{2}$. The correlation of each output variable with $x_{2}$ is comparatively weaker than with $x_{1}$.

**Table 3:** Correlation coefficient between input-output variables.

	$x_{1}$	$x_{2}$	$y^{g}$	$y_{1}^{b}$	$y_{2}^{b}$	$y_{3}^{b}$
$x_{1}$	**1**					
$x_{2}$	**0.78**	**1**				
$y^{g}$	**0.37**	**0.56**	**1**			
$y_{1}^{b}$	**−0.26**	**−0.16**	**−0.12**	**1**		
$y_{2}^{b}$	**−0.18**	**−0.08**	**−0.48**	**0.16**	**1**	
$y_{3}^{b}$	**−0.26**	**−0.09**	**−0.13**	**0.06**	**0.42**	**1**

## 4 Results

### 4.1 Results under SBM-undesirable model

We used the SBM model with undesirable outputs to evaluate fiscal expenditure efficiency in the Target Cities. An efficiency value of 1 indicates effective fiscal expenditure in a Target City. The results are presented in Table B1 of the Appendices. The average efficiency value of the Target Cities from 2017 to 2019 is above 0.5, as shown in Table 4.

**Table 4:** Efficiency in different years.

Efficiency	2017	2018	2019
mean	0.83	0.57	0.67
std	0.26	0.34	0.34

### 4.2 General efficiency analysis

#### 4.2.1 Annual efficiency analysis

We further analyzed the efficiency values of the Target Cities from 2017 to 2019, categorizing them based on whether the efficiency value is 1. The findings are shown in Table 5.

**Table 5:** Annual efficiency of Target Cities.

Year	Efficiency	Number of DMUs	Proportion
2017	ρ=1	8	66.67 %
	ρ<1	4	33.33 %
2018	ρ=1	10	37.04 %
	ρ<1	17	62.96 %
2019	ρ=1	13	48.15 %
	ρ<1	14	51.85 %

In 2017, out of the 12 Target Cities involved in the trial implementation of the clean heating policy, seven cities—Tangshan, Baoding, Langfang, Hengshui (all in Hebei Province), Jinan, Taiyuan, and Kaifeng—achieved fiscal expenditure efficiency of 1. Additionally, Tianjin, the only municipality directly under the central government among the 12 Target Cities, also demonstrated efficiency. Notably, Hebi and Xinxiang, which face severe air pollution and have lower levels of socio-economic development, exhibited lower efficiency in policy implementation. These findings provide empirical evidence that a uniform “one policy for all” approach may disproportionately impact low-income households. Policymakers should consider the distributional effects when designing energy transition policies for a clean and low-carbon economy [32]. Shijiazhuang and Zhengzhou, the provincial capitals of Hebei and Henan Provinces, respectively, also exhibited lower efficiency.

In 2018, all 27 Target Cities participated in the implementation of the clean heating policy. The percentage of efficient Target Cities in 2018 was lower compared to 2017. For example, among the seven Target Cities in Henan Province, only Anyang demonstrated efficiency. Other Target Cities in Henan Province had fiscal expenditure efficiency values below 0.5. Similar to 2017, three out of the four provincial capitals, namely Shijiazhuang, Jinan, and Zhengzhou, exhibited lower efficiency.

The number of efficient Target Cities in 2019 remained relatively stable compared to 2018. In Henan Province, three out of the seven Target Cities achieved efficiency in 2019: Zhengzhou (the provincial capital), Kaifeng, and Jiaozuo. Moreover, three cities in Henan Province—Hebi, Xinxiang, and Puyang—improved their efficiency values in 2019 compared to 2018. This indicates a significant enhancement in fiscal expenditure efficiency in Henan Province during the third year of policy implementation.

Tianjin, Baoding, Langfang, Changzhi, Dezhou, and Heze maintained efficiency throughout the period of 2017–2019, indicating their superior performance in implementing the clean heating policy and achieving air pollutant reduction targets compared to their peer Target Cities. On the contrary, the annual efficiency of Shijiazhuang (the provincial capital of Hebei Province), Zhengzhou (the provincial capital of Henan Province), Hebi, Xinxiang, Puyang (all three in Henan Province), Xingtai, and Handan (both in Hebei Province) remained below average during 2017–2019.

#### 4.2.2 Regional efficiency analysis

**Fig. 1:**
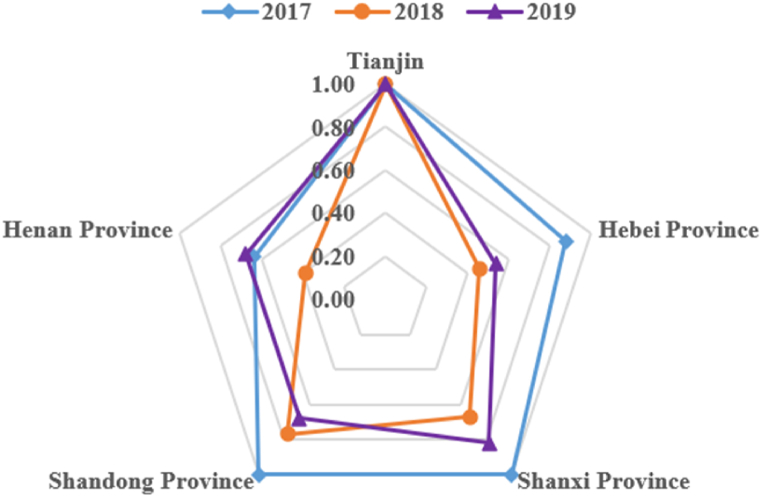
Average efficiency values of five provincial-level administrative regions.

Table 6 and Fig. 1 present a comparison of the fiscal expenditure efficiency among the five provincial-level administrative regions. Tianjin, a provincial-level municipality, exhibited the highest efficiency during the period of 2017–2019. Among the four provinces that house the Target Cities, Henan Province slightly lagged behind the other three in terms of achieving fiscal expenditure efficiency, but it significantly improved its efficiency in 2019. However, the fiscal expenditure efficiency of the other three provinces, i.e., Hebei, Shanxi, and Shandong, exhibited a downward trend.

**Table 6:** Average efficiency of different provincial-level administrative regions.

Average efficiency	2017	2018	2019
Tianjin	1	1	1
Hebei Province	0.88	0.46	0.54
Shanxi Province	1	0.67	0.82
Shandong Province	1	0.77	0.68
Henan Province	0.64	0.39	0.68
Provincial average a	0.88	0.57	0.68

a Provincial average means the average of the efficiency of 26 cities in the four provinces; the municipality directly under the central government (Tianjin) is not included.

Fiscal expenditure efficiency is highest in Tianjin, a municipality directly under the central government. This can be attributed to the city receiving relatively more financial subsidies compared to other Target Cities and benefiting from higher levels of economic development and infrastructure. As a result, the implementation of CCHP in Tianjin has achieved rapid, favorable, and efficient outcomes. Henan Province demonstrates relatively lower overall efficiency. The reasons for this could be its larger population base, lower level of economic development, greater dependence on fossil fuels in production and operations, insufficient fiscal support, and initial resistance against CCHP policy implementation. Consequently, the fiscal funds invested in air pollution control and the promotion of new energy sources did not yield significant results until 2019, when fiscal expenditure efficiency showed improvement. The declining trend observed in Hebei, Shanxi, and Shandong Provinces indicates that the initial CCHP policy implementation was characterized by high intensity, followed by a certain level of fund redundancy in the later stage. It may be appropriate to consider reducing policy subsidies for CCHP in these regions.

**Fig. 2:**
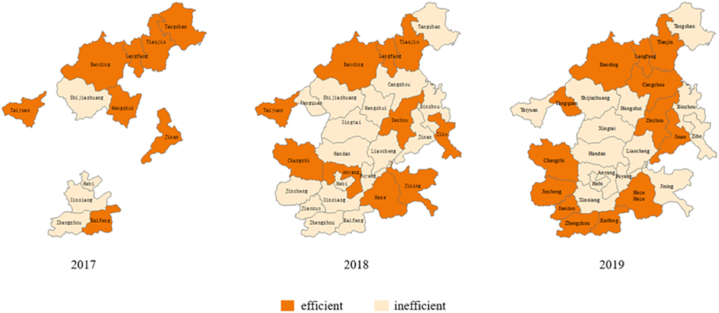
Distribution of efficiency values of Target Cities.

The efficiency values of Target Cities from 2017 to 2019 are spatially represented in Fig. 2. We divided the Target Cities into efficient cities and inefficient ones. We then colored the two groups with orange and beige, respectively. Only a few cities maintained the same color throughout all three years (Fig. 2). Target Cities within the same province generally have synchronized color-changing patterns.

In Hebei Province, Baoding and Langfang maintained efficiency over the entire period, indicating the steady implementation of the clean heating policy in these cities. Cangzhou closely followed suit, achieving efficiency in 2019. Similarly, Changzhi in Shanxi Province, and Dezhou and Heze in Shandong Province maintained efficiency throughout the period of 2018–2019. However, the fiscal expenditure efficiency of other Target Cities fluctuated over the years. For example, Tangshan and Hengshui in Hebei Province experienced a decline in efficiency. This result suggests a certain degree of redundancy in the later stage of the period. On the other hand, in Jiaozuo and Puyang in Henan Province, efficiency increased each year. This indicates that the financial resources for clean heating in Henan Province are gradually being allocated effectively. The full implementation of this policy is expected in the coming years. Overall, different regions and cities exhibited uneven and fluctuating efficiency patterns. This result resonates with a prior study, which found that regional disparities of subsidies under CCHP exacerbated the heating burdens on low-income households and heating poverty in less developed regions [31].

### 4.3 Robustness test

To validate the results and address concerns regarding methodological bias, we conducted a robustness check using the UINP model. As explained earlier in Section 3.1., UINP is another widely used DEA model. This additional analysis aims to ensure the reliability and consistency of our findings. The UINP model incorporates the treatment of undesirable outputs within the standard DEA framework by considering them as inputs [63]. The underlying rationale is to reduce both inputs and undesirable outputs simultaneously. The mathematical formulation of the UINP model is as follows:

$$\rho^{-1}=\max\;\theta$$

(4) $$s.t.\;X\lambda \leq x_{o}$$

$$Y^{g}\lambda \geq {\theta y}_{o}^{g}$$

$${\;Y}^{b}\lambda \leq y_{o}^{b}$$

λ≥0

We present the computational results of the UINP model in Table B2 of the Appendices. We then compare the results obtained from the SBM model with undesirable outputs and the UINP model to determine the relative rankings of each Target City. These comparative results are presented in Table 7. The findings reveal a high degree of similarity between the computational results obtained from the two methods, and the results pass the T test. Importantly, the main conclusions drawn from our analysis remain consistent. This further affirms the robustness of our calculations and strengthens the validity of our findings.

**Table 7:** Relative ranking of fiscal expenditure efficiency of Target Cities under two different models.

City	2017		2018		2019	
	SBM	UINP	SBM	UINP	SBM	UINP
Tianjin	1	1	1	1	1	1
Shijiazhuang	11	11	17	17	20	21
Tangshan	1	1	20	23	24	23
Baoding	1	1	1	1	1	1
Langfang	1	1	1	1	1	1
Hengshui	1	1	21	21	27	27
Handan	–	–	24	26	21	22
Xingtai	–	–	26	25	23	26
Cangzhou	–	–	15	22	1	1
Taiyuan	1	1	1	1	22	17
Yangquan	–	–	14	15	1	1
Changzhi	–	–	1	1	1	1
Jincheng	–	–	23	19	1	1
Jinan	1	1	12	12	1	1
Zibo	–	–	1	1	26	25
Jining	–	–	1	1	14	14
Binzhou	–	–	11	13	25	24
Dezhou	–	–	1	1	1	1
Liaocheng	–	–	18	20	17	18
Heze	–	–	1	1	1	1
Zhengzhou	9	9	13	11	1	1
Kaifeng	1	1	22	18	1	1
Hebi	12	12	19	14	16	15
Xinxiang	10	10	25	24	19	19
Anyang	–	–	1	1	18	20
Jiaozuo	–	–	16	16	1	1
Puyang	–	–	27	27	15	16

## 5 Discussion

### 5.1 Main observations

Our study reveals spatial disparities in the fiscal expenditure efficiency of the Target Cities under CCHP. Provincial capitals and cities with relatively low economic development levels performed poorly. But some cities achieved perfect efficiency each year. This observation suggests that the initial distribution of central government subsidies, based solely on administrative levels, may not be the most efficient approach. It overlooks crucial socio-economic factors, such as resource endowment heterogeneity, economic development levels, pollution levels, and the number of users of coal-fired heating in the city. Our study aligns with Meng et al. [8], which highlights the uneven distribution of benefits and costs under CCHP. This uneven distribution emphasizes the challenges faced by residents in mountainous areas, where they experience fewer benefits and higher costs, resulting in lower cost-effectiveness. Other scholars have also suggested that regional differences should be addressed by providing targeted investment and subsidies in less developed areas to achieve multiple development goals, such as improved air quality, reduced health impacts, and decreased inequity in future clean heating interventions [8,32,64]. Our study emphasizes the importance of addressing disparities and tailoring policies to specific regional needs for more effective and equitable implementation.

Regarding the temporal dimension, achieving perfect efficiency in government subsidies under CCHP at the city level may take several years. Most Target Cities in Henan Province, including Hebi, Xinxiang, Jiaozuo, and Puyang, required the entire three-year study period to show an upward efficiency trend. Some Target Cities, such as Shijiazhuang, Handan, Xingtai, Binzhou, and Liaocheng, did not experience significant efficiency improvement or even saw a decrease during the three-year period. This variation suggests that local conditions and initial infrastructure differences play significant roles in determining efficiency outcomes. Substantial infrastructure projects and behavioral changes in energy consumption typically require extended periods to yield optimal results. Therefore, we might expect the efficiency profiles of the Target Cities to further develop beyond the study period.

Adopting a sweeping approach to phasing out government subsidies might be detrimental to CCHP. Abrupt discontinuance of central government subsidies at the end of the scheduled three years could deplete the financial resources of pilot cities and undermine the objectives of CCHP. Additionally, the hasty implementation of CCHP left several challenges unaddressed, such as insufficient and unstable energy supply, high energy costs, and issues with implementation by local bureaucrats [3]. Addressing these challenges requires ongoing financial support from both central and local governments. Our findings align with Xie et al. [32], which argues for considering the distributional effects when designing energy transition policies.

This study offers three valuable lessons for policymakers and planners when designing and evaluating comparable government subsidies. Firstly, we propose a 17-factor test that accounts for the socio-economic conditions of cities (Table 8). Policymakers and planners are encouraged to incorporate these factors into the design and evaluation of efficient central government subsidies.

**Table 8:** Basis for allocation of central government subsidies under energy policies.

Dimensions	Factors
**Economic**	Per capita GDP
	Per capita disposable income (PCDI)
	Fiscal revenue and expenditure level
**Population**	Urban population density
	Rural population density
	Urbanization rate
**Social**	Clean heating rate
	Building structure
	Number of reconstructed households
	Resident satisfaction
**Resource endowment**	Industrial structure
	Energy-resource structure
**Health and environment**	Average daily temperature in winter (°C)
	AQI
	SO_2_ (μg/m3)
	PM2.5 (μg/m3)
	NOx (μg/m3)

Secondly, we recommend a progressive phase-out of government subsidies under large-scale energy policies like CCHP to improve efficiency. A progressive phase-out should be slow, step-by-step, and well-planned. Policymakers should also incorporate safeguard measures, such as surveying local residents’ preferences and affordability, monitoring, and conducting post-evaluations of the clean heating devices. These measures would allow end users to adapt gradually, understand the benefits of clean energy, and create momentum for successful implementation.

Finally, policymakers and planners should focus on specific areas when assessing the viability of nationally implemented government subsidies. They should evaluate whether local government subsidies are streamlined to increase synergy and scale effects. Currently, there is a lack of unification and standardization in local policies and financial subsidies among the Target Cities. Scale effects can be achieved through coordinated choices of clean energy for nearby cities, which may share infrastructure and human resources [22,23]. Policymakers and planners should also explore creative funding options. For example, they may collaborate with local enterprises and financial institutions to mobilize the market for resource allocation. This option alleviates the pressure on initial local government subsidies and enhances the policy's sustainability.

### 5.2 Policy content analysis of Efficient Six and Inefficient Six

We now engage in a policy content analysis in order to gain a deeper understanding of the city-level managerial efficiency efforts. We collected CCHP-related normative documents of the six best-performing cities (Tianjin, Baoding, Langfang, Changzhi, Dezhou, and Heze, collectively, the Efficient Six) and the six cities with lower efficiency (Shijiazhuang, Xingtai, Handan, Liaocheng, Hebi, and Xinxiang, collectively, the Inefficient Six) based on our findings in Section 4. The normative documents are issued by both the central and the local (city) governments. We searched on PKULAW.CN, the most comprehensive professional database of Chinese law. We retrieved documents from 2017 to 2019 using CCHP key words such as “clean heating,” “coal to electricity,” “coal to gas,” “double substitution,” and “financial allocation.” We collected a total of 124 normative documents. We conducted an inductive reading of the full texts of these documents to qualitatively identify common themes. Additionally, we quantitatively identified high-frequency words unique to the Efficient Six and the Inefficient Six, respectively. We then compared the contents of the local documents with those promulgated by the central government.

Table 9 compares the high-frequency words unique to the Efficient Six and the Inefficient Six, categorized into five themes: “Policy implementation,” “Geographic areas,” “Government bodies,” “Social responsibility,” and “Technical pathways.” The Efficient Six placed a strong emphasis on user initiative by setting high implementation standards, using words that convey a mandatory nature (such as “attaining” and “realizing”), and expressing conscious activity (such as “rigorously”). They also specified specific geographical areas for implementation, identified a limited number of government bodies with clear hierarchies, and provided flexibility in terms of technical pathways. In contrast, the Inefficient Six seemed to view the policy goals as non-mandatory, using words such as “constructing” and “developing.” They endorsed a passive role of users, as indicated by terms like “as specified” and “up to standard.” They also used more general terms when referring to geographic areas for implementation, involved numerous government bodies at the same level, and narrowed down the technical pathways to “coal to electricity” and “coal to gas.” Furthermore, normative documents of the Inefficient Six rarely contained social responsibility expressions.

**Table 9:** Comparison of high-frequency words unique to Efficient Six and Inefficient Six.

	Efficient Six	Inefficient Six
Policy implementation	Attaining	Constructing Developing As specified Up to standard
	Realizing	
	Vigorously	
Geographical areas	Development areas	Districts and counties Countryside
	Demonstration areas	
	Urban districts	
	Well-built areas	
	Rural areas	
Government bodies	Construction Bureau	Construction Committee Quality Supervision Bureau Planning Bureau Urban Management Bureau
	Administration	
	Municipal Party Committee	
Social responsibility	Enterprise participation	(Rarely mentioned)
	Social investment	
Technical pathways	Energy transformation	Coal to electricity Coal to gas
	Thermoelectricity	
	Renewable energy	
	Natural gas	

Moreover, the normative documents of the Efficient Six exhibited a higher degree of consistency with central normative documents. For example, the Efficient Six emphasized the central role of the Construction Bureau, aligning with the central government's emphasis on building energy conservation. Additionally, their open view on technical pathways aligned with the central government's recommendation of using technologically diversified schemes tailored to local conditions.

Overall, the content analysis suggests that the formation of well-designed legal and policy documents may contribute to higher efficiency levels. It is important to align local normative and policy documents with the central design. This helps clarify the policy's mandate and areas, preserve flexibility in implementation, and offer clear guidance to both end-users and government officials. This cohesive approach will facilitate smoother and more effective policy implementation, leading to better outcomes and increased success in achieving the desired objectives.

## 6 Conclusions

CCHP is among the most radically and aggressively implemented energy policies worldwide. We conducted a comprehensive analysis of the fiscal expenditure efficiency under CCHP in 27 pilot cities from 2017 to 2019. Overall, efficiency was commendably high, with an average value exceeding 0.5. Nearly half of the Target Cities were efficient in both 2017 and 2019. However, uneven efficiency profiles were observed, with a decrease in the number of efficient cities compared to the first year of policy implementation. Provincial capitals and economically less developed cities performed poorly, while some cities consistently achieved perfect efficiency.

Our study emphasized that the initial distribution of central government subsidies, based solely on administrative levels, may lead to low financial efficiency. This approach overlooks crucial socio-economic factors such as resource endowment heterogeneity, economic development levels, pollution levels, and the number of coal-fired heating users in each city. Our findings align with prior research highlighting unevenly distributed benefits and costs under CCHP [8]. We also found that better-formulated legal and policy documents in certain cities may have contributed to their higher efficiency. The change in 2021, where the allocation of central government subsidies for CCHP shifted to a competitive evaluation process [18], indicates a positive shift in the Chinese central government's recognition of the importance of fund allocation efficiency. The actual impact and effectiveness of this revised scheme remain to be observed.

Achieving perfect efficiency in government subsidies at the city level might take several years after the initiation of CCHP. Some Target Cities needed the entire three-year study period to show an upward efficiency trend, while others experienced limited improvement or even decreased efficiency. This highlights the need for a more cautious approach to phasing out government subsidies, avoiding abrupt discontinuation after the three-year period. We propose a refined 17-factor test and recommend a progressive phase-out schedule with safeguard measures to ensure the long-term effectiveness of nationally implemented subsidies.

This study has two limitations. First, the choice of indicators was constrained by data availability. Except for Tianjin, All Target Cities are prefecture-level cities where CCHP-related data are considerably limited. This is primarily due to the use of different statistical methods for certain factors or the unavailability of specific data. As a result, our selection of indicators was restricted to a limited number that had accessible and comparable data. Second, the study predominantly adopts a governmental perspective and does not sufficiently consider the perspectives of other stakeholders. Other stakeholders may include interested oil and gas enterprises and local residents. Incorporating a broader range of stakeholders and considering health benefits would contribute to a more comprehensive and objective evaluation.

There are three possible avenues for future research. First, as data becomes available in subsequent years, the cities under study may be expanded from the “2 + 26” key cities to include a majority or all of the 63 pilot cities currently approved to obtain a more complete efficiency picture of CCHP. Second, our content analysis suggests that certain provinces under study provided for a portion of the government subsidies to be awarded subsequently to the best performing cities. Future studies could focus on evaluating provincial and city performance to determine the best performers. Third, with the help of the GIS models and spatial econometric models, future studies may identify the spatial effects and spatial heterogeneity of CCHP. They may study the impact of “economic distance” on the efficiency of policy implementation as well as on the selection of the optimal clean heating pathway.

## Data availability statement

Data will be made available on request.

## CRediT authorship contribution statement

**Fei Mo:** Writing – review & editing, Writing – original draft, Supervision, Resources, Project administration, Methodology, Investigation, Funding acquisition, Formal analysis, Conceptualization. **Yaoyao Ren:** Writing – review & editing, Writing – original draft, Visualization, Validation, Software, Methodology, Formal analysis, Data curation, Conceptualization.

## Declaration of competing interest

The authors declare that they have no known competing financial interests or personal relationships that could have appeared to influence the work reported in this paper.

## Figures and Tables

**Table A1:** Summary of clean heating pilot cities.

Clean heating	City or area	Provinces involved
The first batch of pilot cities (2017)	Tianjin, Shijiazhuang, Tangshan, Baoding, Langfang, Hengshui, Taiyuan, Jinan, Zhengzhou, Kaifeng, Hebi, Xinxiang	Tianjin, Hebei, Shandong and Henan Provinces
The second batch of pilot cities (2018)	Handan, Xingtai, Zhangjiakou, Cangzhou, Yangquan, Changzhi, Jincheng, Jinzhong, Yuncheng, Linfen, Luliang, Zibo, Jining, Binzhou, Dezhou, Liaocheng, Heze, Luoyang, Anyang, Jiaozuo, Puyang, Xi'an, Xianyang	Hebei, Shandong, Henan and Shanxi Provinces
The third batch of pilot cities (2019)	Dingzhou, Xinji, Sanmenxia, Jiyuan, Tongchuan, Weinan, Baoji and Yangling Demonstration Areas	Hebei, Henan and Shanxi Province
The fourth batch of pilot cities (2021)	Beijing, Yantai, Taian, Weifang, Chengde, Datong, Qizhou, Qinhuangdao, Shuozhou, Xuchang, Yulin, Yanan, Lanzhou, Fuxin, Jiamusi, Baotou, Urumqi, Haixi Mongolian and Tibetan Autonomous Prefecture, Liaoyuan, Wuzhong	Beijing, Shandong, Hebei, Shanxi, Henan, Shaanxi, Gansu, Liaoning, Jilin, Heilongjiang and Qinghai Provinces, Inner Mongolia Autonomous Region, Xinjiang Uygur Autonomous Region, and Ningxia Hui Autonomous Region
"2 + 26″ key cities along the pathway through which air pollution in the Beijing-Tianjin-Hebei Region transmits	Beijing, Tianjin, Shijiazhuang, Tangshan, Langfang, Baoding, Cangzhou, Hengshui, Xingtai, Handan, Taiyuan, Yangquan, Changzhi, Jincheng, Jinan, Zibo, Jining, Dezhou, Liaocheng, Binzhou, Heze, Zhengzhou, Kaifeng, Anyang, Hebi, Xinxiang, Jiaozuo, Puyang	Beijing, Tianjin, Hebei, Shanxi, Shandong and Henan Province

**Table A2:** Summary of clean heating technology paths.

Clean heating	Technical path	Technology path subdivision
	Natural gas heating	Gas fired cogeneration
		Gas fired boiler
		Gas distribution
		Wall mounted gas boiler
	Electric heating	Centralized electric boiler
		Centralized heat pump
		Household electric heater
		Household heat pump (including air source, water source and shallow geothermal heat pump)
	Clean coal heating	Ultra-low emission cogeneration
		Large and medium-sized coal-fired boiler
	Renewable energy heating	Geothermal heating
		Biomass heating
		Solar heating
	Industrial waste heat heating	

**Table B1:** Results of efficiency under SBM-Undesirable model.

City	2017	2018	2019
Tianjin	1.00	1.00	1.00
Shijiazhuang	0.39	0.35	0.32
Tangshan	1.00	0.30	0.25
Baoding	1.00	1.00	1.00
Langfang	1.00	1.00	1.00
Hengshui	1.00	0.29	0.21
Handan	–	0.20	0.29
Xingtai	–	0.16	0.26
Cangzhou	–	0.39	1.00
Taiyuan	1.00	1.00	0.27
Yangquan	–	0.44	1.00
Changzhi	–	1.00	1.00
Jincheng	–	0.23	1.00
Jinan	1.00	0.52	1.00
Zibo	–	1.00	0.23
Jining	–	1.00	0.87
Binzhou	–	0.52	0.23
Dezhou	–	1.00	1.00
Liaocheng	–	0.34	0.41
Heze	–	1.00	1.00
Zhengzhou	0.70	0.47	1.00
Kaifeng	1.00	0.28	1.00
Hebi	0.33	0.31	0.44
Xinxiang	0.53	0.19	0.34
Anyang	–	1.00	0.36
Jiaozuo	–	0.37	1.00
Puyang	–	0.07	0.64

**Table B2:** Results of efficiency under UINP model.

City	2017	2018	2019
Tianjin	1.00	1.00	1.00
Shijiazhuang	0.32	0.21	0.18
Tangshan	1.00	0.14	0.14
Baoding	1.00	1.00	1.00
Langfang	1.00	1.00	1.00
Hengshui	1.00	0.15	0.08
Handan	–	0.06	0.17
Xingtai	–	0.09	0.08
Cangzhou	–	0.14	1.00
Taiyuan	1.00	1.00	0.22
Yangquan	–	0.26	1.00
Changzhi	–	1.00	1.00
Jincheng	–	0.16	1.00
Jinan	1.00	0.55	1.00
Zibo	–	1.00	0.09
Jining	–	1.00	0.67
Binzhou	–	0.26	0.10
Dezhou	–	1.00	1.00
Liaocheng	–	0.16	0.20
Heze	–	1.00	1.00
Zhengzhou	0.66	0.63	1.00
Kaifeng	1.00	0.19	1.00
Hebi	0.17	0.26	0.44
Xinxiang	0.35	0.10	0.20
Anyang	–	1.00	0.19
Jiaozuo	–	0.24	1.00
Puyang	–	0.03	0.41

## Appendix A

We summarized the pilot cities in 2017–2021 and technology paths of clean heating policy.

## Footnotes

2 The term “efficiency” can be used interchangeably with the term “effectiveness” in this article.
